# Re-expression of HPV16 E2 in SiHa (human cervical cancer) cells potentiates NF-κB activation induced by TNF-α concurrently increasing senescence and survival

**DOI:** 10.1042/BSR20140160

**Published:** 2015-02-25

**Authors:** Devan Prabhavathy, Chandrasekaran Karthik Subramanian, Devarajan Karunagaran

**Affiliations:** *Department of Biotechnology, Bhupat and Jyoti Mehta School of Biosciences, Indian Institute of Technology Madras, Chennai 600 036, India

**Keywords:** cytokines, E2, human papillomavirus, nuclear factor kappa-light-chain-enhancer of activated B-cells (NF-κB), senescence, survival, tumour necrosis factor (TNF)-α, 4-MUG, 4-methylumbelliferyl-β-D-galactopyranoside, BrdU, bromodeoxyuridine, Cip1, CDK inhibitor p21, cyc D1, cyclin D1, HEK, human embryonic kidney, HMG, high mobility group, HPV, human papilloma virus, HR-HPV, high-risk human papillomavirus, hTERT, human telomerase reverse transcriptase, IL, interleukin, NF-κB, nuclear factor kappa-light-chain-enhancer of activated B-cells, p-RelA, phospho-RelA, qPCR, quantitative real-time PCR, SA, senescence-associated, SASP, senescence-associated secretory phenotype, SMS, senescence messaging secretory, STAT3, signal transducer and activator of transcription 3, TNF, tumour necrosis factor

## Abstract

Re-expression of E2 in human papillomavirus (HPV) transformed tumour cells can induce apoptosis; however, some evidences also attribute an important role to E2 in sustaining tumorigenesis. In the present paper, we studied the effects of tumour necrosis factor (TNF)-α-mediated NF-κB (nuclear factor kappa-light-chain-enhancer of activated B-cells) activation on E2-induced senescence in HPV16-integrated SiHa cells. The results show that E2 inhibits endogenous E6 gene expression and sensitizes SiHa cells to TNF-α-induced NF-κB activation. Under this condition there was an increase in the expression of senescent proteins p53, p21, p27 and p16 and senescence-associated (SA)-β-galactosidase activity indicating that TNF-α augments E2-mediated senescence. Re-expression of E2 expression with TNF-α treatment resulted in an increase in the expression of anti-apoptotic Bcl2 (B-cell lymphoma 2) protein and other pro-survival genes like cyclin D1 (cyc D1), survivin and hTERT (human telomerase reverse transcriptase). Concomitantly, E2 + TNF-α combination increased the survival of SiHa cells by positive changes in viability, proliferation and colony formation. E2-induced apoptotic tendency shifted towards senescence in presence of TNF-α by arresting the cells at both G_0_/G_1_ and G_2_/M phases, thus enhancing cell survival. Another observation in the present study is the significant up-regulation of key senescence messaging factors regulated by NF-κB namely interleukin (IL)-6, IL-8, high-mobility group protein A (HMGA)1 and B (HMGB)1 in E2-transfected cells treated with TNF-α. Our data provide a mechanistic basis and a new insight for the role of TNF-α and E2 in linking cellular senescence, tumorigenesis and HPV re-infection.

## Abstract 2

Human papillomavirus (HPV)16 E2 potentiates NF-κB (nuclear factor kappa-light-chain-enhancer of activated B-cells) activation induced by tumour necrosis factor (TNF)-α in SiHa (human cervical cancer) cells and significantly influences cell viability, apoptosis and expression of pro-survival genes regulated by NF-κB.

## INTRODUCTION

Cervical neoplastic progression is characterized by high-risk human papillomavirus (HR-HPV) infection followed by viral integration and episome clearance [1]. Early viral gene expression (E1, E2, E4, E5, E6 and E7) plays an important role in host cell survival irrespective of the viral genome being episomal or integrated [2]. HPV E2 is one of the early genes known to repress the expression of E6 with a consequent increase in p53 levels [3]. Loss of E2 expression during HR-HPV integration into the host genome results in the constitutive activation of the viral oncogenes, E6 and E7 [4]. Besides, E2 induces growth arrest in the G_1_-phase of the cell cycle [5] and induces apoptosis through the activation of caspase 8 [6]. In contrast, sustained p53 activation subsequent to E2-mediated DNA damage can also promote pro-tumorigenic inflammation and senescence [7]. Interestingly, depletion of viral early genes including E2, up-regulates genes involved in autophagy and exhibits senescence-associated secretory phenotype (SASP) [2] implicating a possible dichotomous role for E2 as an oncogene and a tumour suppressor in HPV-induced carcinogenesis. Re-infection with HR-HPV [8] together with the presence of other cofactors of cervical carcinogenesis like tobacco smoking, oral contraceptives, multiparity and other sexually transmitted infections together with chronic inflammation, can provide a conducive environment for extended DNA damage [9]. Such changes could confer a selective advantage for an individual cell and ultimately contribute to carcinogenic progression. Apart from these, cell-mediated immune responses greatly influence the development of neoplasia by HR-HPV. Subsequent events and their associated cellular changes result in chronic inflammation by converging at the level of an important transcription factor, NF-κB (nuclear factor kappa-light-chain-enhancer of activated B-cells) [10]. For instance, expression of tumour necrosis factor (TNF)-α is enhanced in response to viral infection and it acts as a key mediator for the regression of HPV-induced lesions [11] by exerting a cytostatic effect on normal and HPV16 immortalized keratinocytes [12,13].

A previous study has shown that HPV E2 can potentiate NF-κB activation by TNF-α [14] and results from our laboratory show that HPV16 E2-mediated potentiation of NF-κB activation induced by TNF-α involves parallel activation of STAT3 (signal transducer and activator of transcription 3) with a reduction in E2-induced apoptosis in epithelial cells [15]. Hence, in the present study, it was of interest to know about the functional outcome of the re-expression of E2 and TNF-α treatment in SiHa (human cervical cancer) cells, considering the role of NF-κB, a major regulator of inflammation, proliferation/survival and DNA damage-induced senescence [16]. Re-introduction of E2 into HPV-associated cervical carcinoma cells, resulting in reactivation of p53 and pRB (phosphorylated retinoblastoma protein) pathways, has been shown to suppress cellular growth, because of cell cycle arrest in G1, apoptosis and senescence [17–20]. The present study provided an insight into the effects of HPV16 E2 re-expression in presence of an inflammatory factor, TNF- α, in cervical cells with specific cellular consequences that led to cell survival.

## EXPERIMENTAL

### Cells, plasmids, transfection and antibodies

The human cervical cancer cell line SiHa was cultured in Dulbecco's modified Eagle's medium (DMEM), supplemented with 10% FBS, 100 mg/ml streptomycin, 100 units/ml penicillin at 37°C with 5% CO_2._ pCMV–E2 (E2 expression plasmid containing cytomegalovirus promoter) (Dr Lawrence Banks, ICGEB, Italy) and pGL4.32–NF-κB–Luc (Promega) plasmids were used. SiHa cells were transfected using Lipofectamine 2000 (Invitrogen) as per manufacturer's protocol. Antibodies to phospho-RelA (p-RelA) (phosphorylated Rel-like domain-containing protein A), p21, Bcl2, p27Kip1 (cyclin-dependent kinase inhibitor p27) and high-mobility group protein A (HMGA)1 (93H1, 2941, 2774, D69C12 and D6A4 respectively, from Cell Signaling), RelA, HPV16 E2, p53 and HMG-1 (9E10, TVG261, 126 and W-18, respectively, from Santa Cruz), p16INK4a (cyclin-dependent kinase inhibitor 2A) (1963, Epitomics), β-actin (A2228, Sigma–Aldrich) and horseradish peroxidase (HRP)-conjugated secondary antibodies (Jackson ImmunoResearch Laboratories) were procured. Human recombinant-TNF-α (PeproTech), resazurin (Himedia) and PI (propidium iodide) (Invitrogen) were also used in the present study.

### Reporter luciferase assays

SiHa cells were seeded in 96-well plates (2.5 × 10^4^) and transiently transfected and treated appropriately. As per manufacturer's protocol, the cell lysates were used to assess the firefly and *Renilla* luciferase activities using specific substrates (Promega dual luciferase kit) and then the luminescence was measured using the SpectraMax L Luminometer.

### Quantitative real-time PCR

Gene specific quantitative SYBR green quantitative real-time PCR (qPCR) assays were performed to monitor expression levels of different genes in Mastercycler® ep realplex^4^ thermal cycler (Eppendorf software version 2.2) using 2× Sensimix low Rox (Roche) by using cDNA reverse-transcribed from RNAs isolated from the SiHa cells transfected and treated appropriately as mentioned. The primers used are listed in the Supplementary Table S1. The quantification was assessed by calculating the 2^(−∆∆^*^C^*_T_^)^ (∆*C*_T_=*C*_T_ target − *C*_T_ reference and *C*_T_ is the threshold cycle value for each of them). ΔΔ*C*_T_=Δ*C*_T_ gene transfected sample − Δ*C*_T_ control transfected sample. The relative quantification of gene expression (target) using β-actin as an internal control (reference) was calculated.

### Immunoblotting

SiHa cells were seeded (1.6 × 10^6^) in 60 mm dishes, transfected with appropriate treatment with or without TNF-α (20 ng/ml) for 24 h. Total lysates using radioimmunoprecipitation assay (RIPA) buffer were prepared and immunoblotted for protein expression studies using specific primary antibodies and appropriate secondary antibodies. Immunoblots were developed by ECL (enhanced chemiluminescence) kit (Amersham).

### SA-β-galactosidase assay

Senescence-associated (SA)-β-galactosidase activity at pH 6.0 was estimated using its substrate, 4-methylumbelliferyl-β-D-galactopyranoside (4-MUG) that does not fluoresce until cleaved by the enzyme to generate the fluorophore 4-methylumbelliferone (4-MU). Cells were processed as described elsewhere [21] without the other modifications.

### Resazurin reduction assay

SiHa cells transfected for 24 h with control or E2 plasmids and treated with TNF-α (20 ng/ml for 24 h) were re-seeded in 96-well plates (0.1 × 10^5^ cells) and incubated for different time durations till assayed. Resazurin dye was added at 10% volume (10 μl of 1 mg/ml) to each well and incubated till a purple colour developed. The supernatant from each well was then diluted 20 times and the amount of resazurin reduced by the cells was determined by measuring its absorbance at 570 nm with 595 nm as background reference using a plate reader (Bio-Rad laboratories) along with a blank control (medium alone). The cell viability was calculated against the untreated control and represented as percentage viability.

### BrdU cell proliferation assay

Cells 24 h, post transfection and treatment with TNF-α (24 h) were reseeded in 96-wells at a density of 15000 cells/well in 100 μl culture medium. The cells were pulsed with bromodeoxyuridine (BrdU) 12 h before the end point of the assay and controls that did not receive the BrdU label. Then cells were assayed for BrdU incorporation using Abcam assay kit as per manufacturer's protocol. The absorbance was read at 450/550 nm, which is directly proportional to the amount of BrdU incorporated into the actively proliferating cells, calculated as percentage against those cells that did not receive BrdU for each sample.

### Annexin V/PI staining for apoptosis detection

After appropriate experimental conditions, around 10^5^ cells were used for analysis. Cells were suspended in 300 μl of 1× binding buffer and incubated with 5 μl of Annexin V–FITC and 5 μl of PI (1 μg/ml) at room temperature (RT) for 5 min as per manufacturer's protocol (Invitrogen). A minimum of 10000 events was analysed by flow cytometer (Beckman Coulter) using the Quanta SC–MPL software program. Annexin V and PI positive cells were counted by using appropriate controls and after compensation of the voltage gating. Data are represented as percentage of cells positive for Annexin V or PI along with the live population for every 10000 cells counted.

### Cell cycle analysis

SiHa cells (1.0 × 10^6^) seeded in six-well plates and appropriately transfected and treated were further trypsinized, washed with ice-cold 1× PBS and fixed overnight in 70% ethanol in PBS–EDTA at −20°C. Cells were then washed with PBS–EDTA containing 1% FBS, suspended in PBS–EDTA and incubated with RNase A (0.2 mg/ml) for 30–45 min at 37°C. DNA staining was done with PI (1 mg/ml) for 15 min. Cells (∼1 × 10^4^) were run on a flow cytometer (excitation: 488 nm laser, emission: 610BP filter) and analysed by the Quanta SC-MPL software (Beckman Coulter) to determine the percentage of cells in G_0_/G_1_, S or G_2_/M phases of the cell cycle.

### Clonogenic cell survival *in vitro*

Cells were transfected and treated appropriately and reseeded in six-well plates (100 cells/well). After 15 days of incubation, the colonies were stained with 0.5% crystal violet made in methanol, washed in running water, air-dried and then counted manually. The number of colonies was counted on an inverted-stage microscope at 40× magnification.

### Statistical analysis

Statistical tests were carried out by unpaired Student's *t* test using GraphPad Prism version 6 software.

## RESULTS

### Re-expression of E2 potentiates TNF-α-induced NF-κB activation and inhibits E6 gene expression in SiHa cells

**Figure 1:**
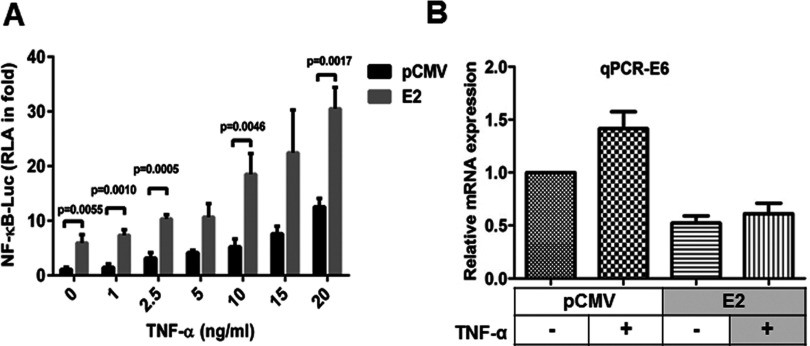
Ectopic expression of E2 potentiated TNF-α-induced NF-κB activation (**A**) SiHa cells were transfected with NF-κB-Luc and pRL–TK (Renilla luciferase vector containing the herpes simplex virus thymidine kinase promoter) plasmids, control (pCMV) or expression plasmids (pCMV–E2) in a combination (expression vector: NF-κB–luciferase construct–*Renilla* luciferase construct=4:1:1) for 24 h, after which, different concentrations (as indicated) of TNF-α treatment was given for 6 h. Then cells were lysed and analysed for firefly luciferase activity that was normalized to *Renilla* luciferase activity in the cell lysates (*n*=3, mean±S.E.M.). (**B**) From cells transfected and treated with TNF-α (20 ng/ml for 6 h) as mentioned above, total RNA was isolated and then subjected to qPCR, for relative quantification of E6 mRNA expression which was normalized against β-actin (*n*=3, mean±S.E.M.). Significance is denoted by *P* ≤ 0.05.

To understand the effect of E2 on TNF-α-mediated NF-κB activation, pCMV vector or E2-transfected SiHa cells were treated with different concentrations of TNF-α (1, 2.5, 5, 10, 15 and 20 ng/ml for 6 h) and analysed for NF-κB luciferase activity. E2-transfected SiHa cells showed a significant induction of around 3-fold in NF-κB transcriptional activity whereas a progressive and significant increase was observed in E2-transfected cells treated with 5 to 20 ng/ml of TNF-α (Figure 1A). Since more than 20 ng/ml of TNF-α caused cell death (result not shown) 20 ng/ml was chosen as an optimal concentration for further studies to elicit maximum response in these cells. To confirm the re-expression of E2, a known regulator of E6 transcription, E6 transcript levels were analysed by qPCR in SiHa cells and as indicated in Figure 1(B), endogenous E6 mRNA levels in SiHa cells were reduced to ∼0.5-fold upon E2 transfection compared with pCMV vector-transfected cells without E2 expression. TNF-α treatment enhanced the endogenous E6 level by ∼0.5-fold as was previously reported [22] and this increase by TNF-α was found to be reduced to ∼0.5-fold by re-expression of E2. These results suggest that re-expression of E2 in SiHa cells inhibited endogenous E6 gene expression, activated NF-κB and interestingly sensitized the cells towards TNF-α-induced NF-κB activation even at sub-optimal doses.

### Potentiation of TNF-α-induced NF-κB activation by E2 up-regulates E2-induced senescence

**Figure 2:**
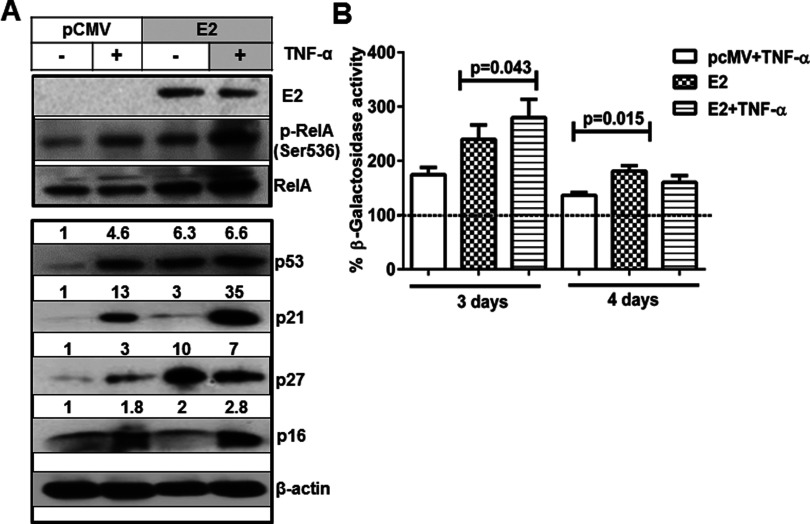
TNF-α-induced NF-κB activation enhanced E2-induced expression of senescent markers (**A**) Whole cell lysates extracted from SiHa cells transfected with pCMV or E2 plasmids (24 h) followed by treatment with TNF-α (20 ng/ml for 24 h) were subjected to immunoblotting studies for the detection of HPV16 E2, p-RelA (Ser536), RelA, p53, p21, p27, p16 and β-actin. Similar results were obtained in another independent experiment. (**B**) Transfected cells (24 h) as mentioned above were treated with TNF-α for 24 h and after another 24 h (3 days) or 48 h (4 days), lysates were analysed for SA β-galactosidase activity using its substrate (4-MUG) in a fluorimeter with an excitation 360 nm and emission wavelength of 465 nm respectively. The enzyme activity was calculated after normalization against total protein of the lysates and expressed as percentage activity relative to the control-transfected cells. Significance is denoted by *P* ≤ 0.05.

Since E2 re-expression in SiHa cells is known to induce senescence [19] it was of interest to analyse the expression of senescent markers in the presence of TNF-α in E2-transfected cells. We transfected SiHa cells with pCMV or E2 expression plasmid followed by treatment with TNF-α (20 ng/ml for 6 h) and the immunoblots in Figure 2A show that E2-transfected cells (E2 expression confirmed by immunoblot) had higher levels of p53 (∼6-fold) and p27 (10-fold) due to down-regulation of E6 [23] and E7 transcription [24] respectively. As expected, p53-regulated p21 (3-fold) and p27-regulated p16 (2-fold) expression levels were increased in E2-transfected cells though the increase in p16 was not significant when compared with the increase in p27 levels. In addition, the increase in p-RelA (Ser^536^) levels was (Figure 2A) consistent with E2-mediated increase in NF-κB transcriptional activity (Figure 1A). Cells treated with TNF-α also increased p-RelA expression though not compared with its increase induced by a combination of E2 and TNF-α (Figure 2A). TNF-α treatment could enhance p53 (∼5-fold), p21 (13-fold), p27 (3-fold) and p16 (∼2-fold) protein levels compared with untreated pCMV vector-transfected cells (Figure 2A). An interesting observation is that E2-transfected cells treated with TNF-α exhibited a tremendous increase in p21 expression (35-fold) with a marginal increase in p27 (7-fold) and p16 (∼3-fold) (Figure 2A). The effect on cellular senescence was further confirmed by assessing SA-β-galactosidase activity. On day 3, post transfection, SA-β-galactosidase activity was higher in TNF-α treated cells (∼0.8-fold increase) and E2-transfected cells (∼1.4-fold increase) and the E2-transfected cells treated with TNF-α, enhanced it further by ∼0.5-fold (∼2-fold) as compared with control (Figure 2B). On day 4, there was a huge dip in the β-galactosidase activity of E2-transfected cells that may be due to loss of expression of E2 known to happen under the conditions of transient transfection. Together these results indicate that E2 augmented the TNF-α-induced NF-κB activation concurrent with a reinforced effect on E2-induced senescence.

### E2-mediated potentiation of TNF-α-induced NF-κB activation increases viability and survival

**Figure 3:**
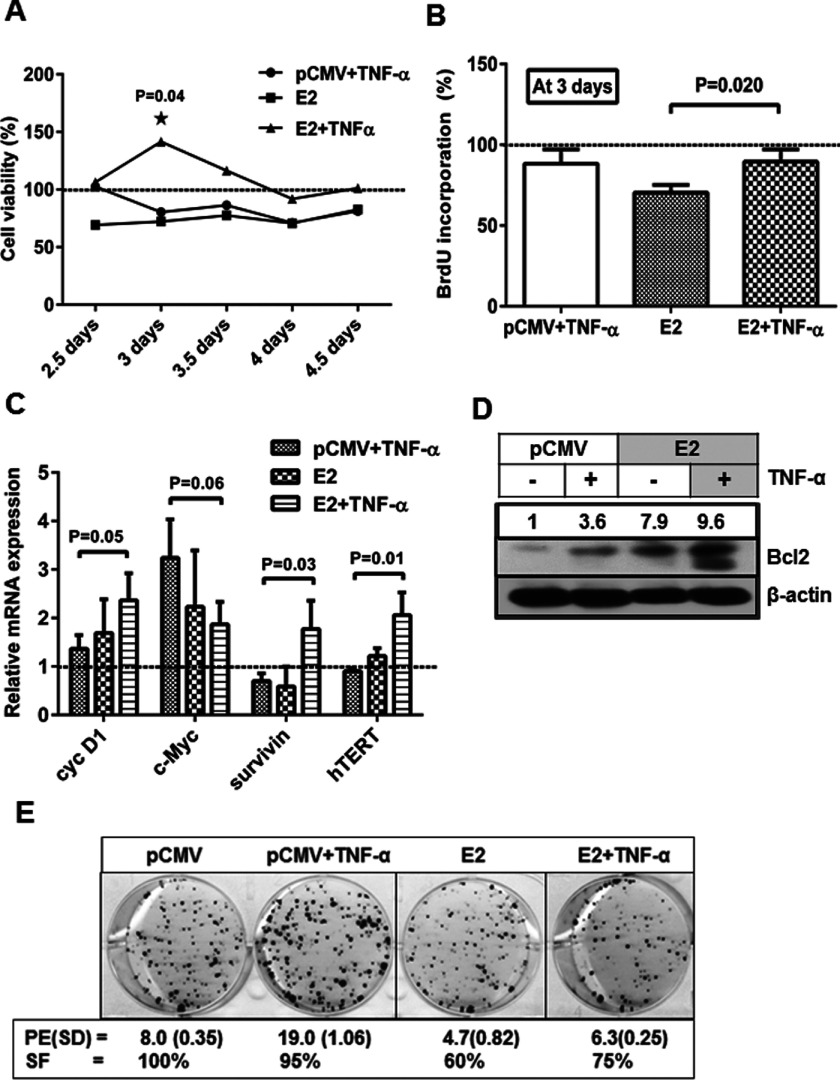
Re-expression of E2 and TNF-α induction increased cell viability and expression of pro-survival genes (**A**) SiHa cells transfected with pCMV or E2 plasmids were assessed for viability using resazurin reduction method and was expressed as percentage against control at indicated days and normalized to seeding density (*n*=3, mean±S.E.M.). (**B**) Cells, transfected and after appropriate treatment, as mentioned above, were assayed for the amount of BrdU incorporation after pulsing for 12 h with BrdU at day 3. The values are expressed as percentage of control (*n*=3, mean±S.E.M.). (**C**) Total RNA was isolated from SiHa cells, 2 days after transfection with pCMV or E2 plasmids and were subjected to qPCR, for relative quantification of expression of cyc D1, c-Myc, survivin and hTERT and expressed in folds relative to control after normalization against β-actin (*n*=3, mean±S.E.M.). Significance denotes *P* ≤ 0.05. (**D**) Cells, transfected and/or treated with TNF-α, as indicated, were subjected to immunoblotting for the expression of Bcl2 protein using the total lysates that were used in Figure 2(A). Similar result was obtained from another independent experiment. (**E**) Transfected and/or TNF-α treated SiHa cells were trypsinized and re-seeded (2000 cells/well) in six-well plates for 15 days. The colonies stained with crystal violet were counted and the plating efficiency (PE, percentage of cells able to form colonies) and survival fractions were calculated (refer Materials and Methods) and mentioned with S.D.. Colony magnifications were photographed at 20×. Plating efficiency was calculated by taking the ratio of the number of colonies formed in the gene transfected plate to that counted in the control whereas survival fraction in percentage indicates the ratio of the plating efficiency in the gene transfected to that of the number of cell seeded.

To study the influence of E2-mediated NF-κB activation on cell viability and survival, several experiments were carried out. TNF-α treatment or E2-transfection decreased the viability of SiHa cells by 20% to 40% compared with control when assessed by the resazurin reduction assay (Figure 3A). In contrast, E2-transfected cells treated with TNF-α showed no such decrease in viability but for a significant increase in viability (40%) compared with control on day 3 (Figure 3A). A concomitant analysis on active DNA synthesis in terms of BrdU incorporation was done at day 3 post transfection and E2-transfected cells showed around 30% decrease whereas TNF-α treatment showed 12% decrease in proliferation compared with control but E2 + TNF-α combination showed ∼20% recovery in proliferation (Figure 3B) indicating that this proliferative/survival ability might be due to E2 + TNF-α-mediated NF-κB activation. Hence, the expressions of pro-survival genes (downstream targets of NF-κB) were analysed. The expression of cyc D1 and c-Myc in cells treated with TNF-α showed around 1.4-fold and 3-fold increase respectively, whereas both were increased by ∼2-fold in E2-transfected cells (Figure 3C). The increase in pro-survival gene expression was correlated with the extent of decrease in cell viability observed in cells treated with TNF-α in the present study (Figures 3A and 3B). TNF-α-mediated STAT3 activation could have contributed for the expression of survival factors like Bcl2 [25] and we have recently shown that STAT3 activation by TNF-α is also potentiated by E2 [15]. Either with E2 transfection or TNF-α treatment, survivin and hTERT mRNA expression rather showed a decrease (Figure 3C) whereas E2 expression together with TNF-α treatment increased the survivin and hTERT transcript levels (2-fold) in addition to cyc D1 and c-Myc (Figure 3C), further supporting the enhanced survival in presence of E2 + TNF-α combination in SiHa cells.

To understand the NF-κB-mediated survival of E2-transfected cells in presence of TNF-α, the expression of an important anti-apoptotic protein Bcl2, was analysed by immunoblotting under such conditions. Although TNF-α treatment could enhance Bcl2 expression by 3.6-fold, E2 expression showed an increase up to 8-fold and their combination further augmented this increase to ∼10-fold in SiHa cells (Figure 3D) indicating that increased Bcl2 expression may be due to NF-κB activation induced by either TNF-α, E2 or their combination as Bcl2 is a well-known downstream target of NF-κB [26]. Clonogenicity assays showed that 95% fraction of cells treated with TNF-α survived though this increase was not greater to the extent as expected based on the increase in the expression of pro-survival genes (Figure 3C). E2-transfected cells showed only 60% of surviving fraction but it increased in presence of TNF-α (75%; Figure 3E) further confirming the survival abilities of E2-expressing cells in presence of TNF-α. The respective plating efficiency is given for each condition and E2 + TNF-α treated cells showed a higher plating efficiency than E2 alone (Figure 3E). Taken together, these results suggest that E2 + TNF-α combination increased the survival of SiHa cells by positive changes in viability, proliferation, expression of pro-survival genes and colony formation.

### TNF-α regulates E2-induced apoptosis by arresting the cells in G_0_/G_1_ and G_2_/M phases

**Figure 4:**
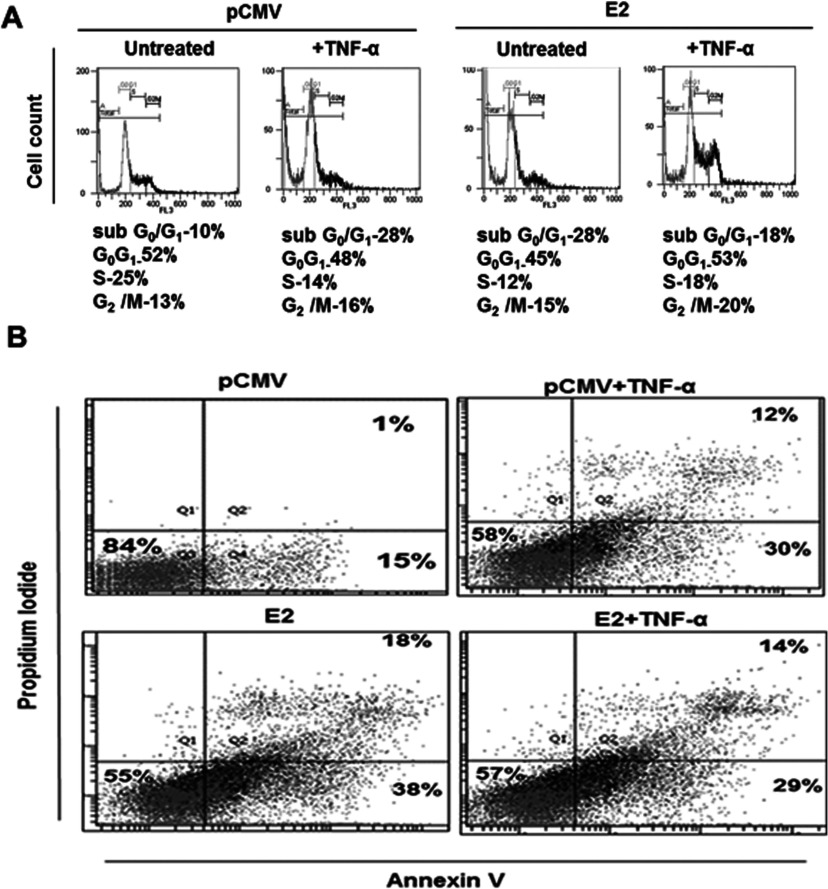
E2 and TNF-α combination reduced apoptosis and increased G_0_/G_1_ arrest pCMV or E2 transfected SiHa cells (24 h) were either given TNF-α treatment for another 24 h (day 2) or left without treatment. (**A**) Transfected and appropriately treated cells were analysed for Annexin V or PI staining to mark the early or late apoptotic populations (mean percentage population respectively, *n*=3. Gating was done using appropriate controls and 10000 cells were considered for each run. Lower-most left quadrant shows the live population whereas the upper right quadrant shows the late apoptotic (PI positive) and the lower right quadrant shows the early apoptotic population (Annexin V positive). (**B**) Under similar experimental conditions, changes in cell cycle phases were studied at a later time, on day 3, by PI staining of the fixed cells and the representative histogram depicts different phases of cell cycle (panel below shows the mean percentage population of different phases, *n*=3).

To see if the changes in survival could be reflected in the extent of apoptosis induced by TNF-α and E2 individually or in combination, Annexin V/PI staining at day 2 post-transfection and treatment was done. TNF-α treatment alone showed an early apoptotic population of 30% and E2-transfected cells with the highest of about 38% whereas their combination reduced the E2-induced effect to 29% (Figure 4B) with no significant difference seen in both live and late apoptotic population among them in cells (both pCMV and E2-transfected) without TNF-α treatment. E2 is known to cause G_2_/M arrest followed by apoptosis [27] and hence, to understand if cell cycle is affected at later time point by the E2 + TNF-α combination, studies on cell cycle were carried out at day 3 of the experiment. Cell cycle analysis showed that TNF-α and E2 increased sub-G_1_ population to 22% and 28%, respectively compared with control (10%) along with the G_2_/M population of 16% and 20% respectively (Figure 4B). In SiHa cells, rather the E2 + TNF-α combination significantly brought down the apoptosis induced by E2 (28%) or TNF-α (22%) to 18% but with a noticeable increase in G_0_/G_1_ population (53%) compared with either E2 (45%) or TNF-α (48%) alone (Figure 4B). Further, G_2_/M arrest was relatively more in E2-transfected cells treated with TNF-α (20%) than E2-transfected (15%) or TNF-α treated (16%) or control cells (13%) alone (Figure 4B). These observations are expected regarding G_2_/M arrest but for the prominent G_0_/G_1_ accumulation shown by E2 + TNF-α combination. G_0_/G_1_ arrest is one of the marked cell cycle features of senescent cells and thus our results indicate that E2 + TNF-α combination resulted in enhanced survival by forcing cells towards senescence. The extended observation at day 4 showed that cell populations in either G_2_/M or sub-G_1_ phases in E2-transfected cells with or without TNF-α treatment found doubled with a stressed morphology indicating the experimental limitation. However, overall results support that E2-induced apoptotic tendency was shifted towards senescence in presence of TNF-α by arresting cells at both G_0_/G_1_ and G_2_/M phases, thus enhancing cell survival.

### Augmentation of E2-induced senescence by TNF-α-induced NF-κB activation leads to the survival of SiHa cells

**Figure 5:**
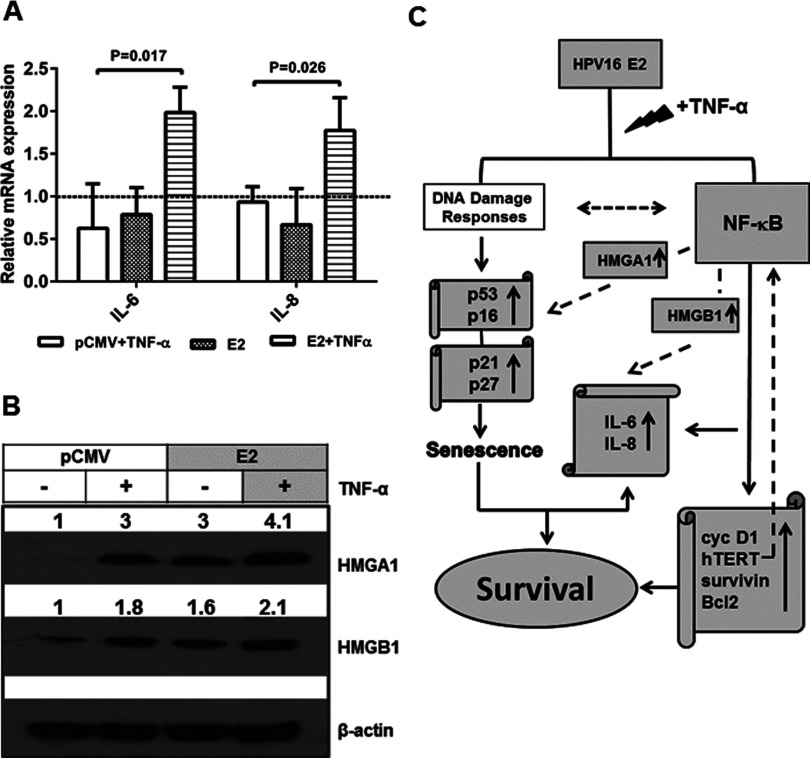
Effect of E2 re-expression and TNF-α treatment on important senescence messaging factors with a mechanistic basis as depicted in the flowchart (**A**) RNAs were harvested from control or E2-transfected cells with or without TNF-α treatment at day 2 and subjected to qPCR for relative quantification of IL-6 and IL-8 transcripts that were normalized to β-actin and the fold change in expression was calculated against the control (*n*=3, mean±S.E.M.). Significance is denoted by *P* ≤ 0.05. (**B**) Whole cell lysates extracted from SiHa cells transfected with control or E2 plasmids (24 h) were subjected to immunoblotting for the detection of HMGA1, HMGB1 and β-actin, with (24 h) or without TNF-α treatment. Similar results were obtained in another independent experiment. (**C**) In the flowchart, HPV16 E2 is a senescence-inducing stimulus, which might activate the DDR which in turn is known to co-operate with NF-κB (indicated by broken line). On the other side, we demonstrated a mechanism (indicated by grey shaded boxes) where HPV16 E2, in response to TNF-α stimulus potentiated NF-κB activation which, in co-operation with HMGA1 led to increased expression of pro-survival factors (cyc D1, hTERT, survivin and Bcl2), pro-senescent proteins (p53, p21, p27 and p16) leading to SASP resulting in enhanced expression of senescence messaging cytokines (IL-6, IL-8), in co-operation with HMGB1, altogether resulting in enhanced cell survival. In turn IL-6, IL-8 and hTERT are known to regulate NF-κB (indicated by broken line).

To further investigate survival/protumoural factors, we analysed the expression of factors functionally associated with NF-κB and demonstrated to be crucial to senescence messaging. The mRNA expression of interleukin (IL)-6 and IL-8 in TNF-α-induced cells or E2-transfected cells did not show any great difference as compared with control cells (both showed a decrease between ∼0.1- to 0.4-fold compared with 1; Figure 5A) whereas E2-transfected cells treated with TNF-α showed an increase in IL-6 and IL-8 at least 1.5-fold in both compared with E2 alone (Figure 5A). Although TNF-α treatment or E2 transfection alone resulted in an increase in HMGA1 protein expression by 3-fold compared with control cells, a combination of E2-transfection and TNF-α treatment increased HMGA1 at least 4-fold. HMGB1 protein expression increased by at least 1.5-fold upon either TNF-α treatment or E2 transfection whereas their combination brought it up to 2-fold. Thus it may be concluded that E2-potentiated TNF-α-induced NF-κB activation enhances the expression of important senescence messaging secretory (SMS) factors, IL-6, IL-8 and HMGB1 and important SASP component HMGA1 and thus providing a mechanistic basis for the observed increase in cell survival.

## DISCUSSION

Our recent observation that HPV16 E2 potentiated TNF-α-induced NF-κB activation and reduced the E2-mediated apoptotic effect in human embryonic kidney (HEK)293 cells [15], with a parallel activation of STAT3 prompted us to understand the effect of re-expression of E2 in HPV16 integrated cervical cancer cells. Enhanced p53 protein expression by E2 re-expression correlates with the observed inhibition of E6 transcription since E6 is well known to induce the degradation of p53 [28]. E2 mediates p53-dependent apoptosis [29] and the C-terminus of E2 is known to interact with p53 leading to differentiation [30]. Induction of p53 results in the transcriptional activation of the cyclin-dependent kinase (CDK) inhibitor p21 (Cip1/Waf1) and promotes cell cycle arrest [31]. E2-mediated potentiation of NF-κB activation induced by TNF-α with a concurrent increase in the expression of p21 in SiHa cells is consistent with the suggested role of NF-κB in TNF-α-mediated cytoprotective effect via the activation of p21(Cip1/Waf1) [32]. Interestingly, Basile et al. [33] reported that p53-independent growth arrest occurs in TNF-α treated human foreskin keratinocytes with increased expression of p21 and differentiation markers. Further, a significant increase in p-RelA levels with a concomitant increase in p21 (Figure 2A) indicated a synergistic NF-κB activation due to the combined effect of E2 and TNF-α, in addition to increased senescence. However, the observed activation of basal levels of NF-κB activation by E2 in SiHa cells suggests the existence of unexplored TNF-α-independent mechanisms regulating NF-κB. Interaction between p300, RelA and E2 observed by us earlier in HEK 293 epithelial cells showing NF-κB activation by E2 even in the absence of TNF-α is one of the plausible mechanisms [15]. p16INk4a expression is usually high in cervical cancers, where the up-regulation is due to the feedback mechanism due to the disruption of RB (retinoblastoma) pathway [34]. Silencing of E6 or/and E7 oncogenes in SiHa cells either by antisense targeting or by re-expression of E2 is known to induce senescence [17,35].

Cell sensitivity to TNF-mediated apoptosis is largely regulated by several factors including viral infections and NF-κB activation finds a critical role in regulating TNF-α sensitivity in cells [36] by regulating the expression of several anti-apoptotic genes. Increased tumorigenicity of HPV16-transformed human keratinocytes is also associated with TNF-α resistance [37], which is acquired during HPV-mediated pathogenesis though the molecular basis is not clear. In the present study, the intended cytoprotective effect of TNF-α-mediated activation of NF-κB, due to increased p21 expression in E2-transfected cells correlated well with a reversal of E2-mediated apoptosis as shown by increase in viability and proliferation. Considering the complexities of NF-κB-mediated functions in response to various genotoxic stimuli (in the present study, E2) the outcome on cell survival is determined by the balance of a pool of anti-apoptotic and pro-survival gene expression [38]. It is relevant that a parallel increase in the expression of anti-apoptotic Bcl2 protein and other pro-survival genes like cyc D1, survivin and hTERT, occurred since they are the targets of NF-κB activation. Interestingly, two recent studies have highlighted the role of TERT in the regulation of NF-κB and vice versa [39,40]. Altogether these changes might have influenced the shift from E2-induced apoptosis to senescence as indicated by increased G_0_/G_1_ and G_2_/M arrest in presence of TNF-α. Our observations showing increased TNF-α-induced apoptosis with high c-Myc gene expression are in agreement with an earlier demonstration suggesting that c-Myc sensitizes cells to TNF-α-induced apoptosis [41]. But SiHa cells treated with TNF-α did not show reduction in apoptosis in contrast with a report in which HPV16 E6 was shown to block TNF-α-induced apoptosis [42]. However, decreased c-Myc gene expression together with increased Bcl2 protein in E2-transfected cells treated with TNF-α showing reduction in TNF-α-induced apoptosis are in accordance with the earlier reports [36,41,43]. Further, an increased surviving fraction of cells that could form colonies as shown by E2-transfected cells treated with TNF-α, supports the continued effect of TNF-α-mediated cell survival.

A new observation in the present study is the significant up-regulation of key SMS cytokines regulated by NF-κB which in turn reinforce senescence growth arrest, namely IL-6 and IL-8 [44] in E2-transfected cells treated with TNF-α, after 2 days, post transfection. Anyhow, SASP is not a transient development; however a low-level DDR (DNA damage response) signalling can bring in SASP [45]. In HR-HPV infections, centrosome amplification and chromosomal abnormalities are detected not only in transformed cancerous cells but also in premalignant lesions [46,47] where the presence of E2 is unambiguous in precancerous lesions and in HPV-transformed cancer cells where re-infection can occur. E2 being a genotoxic molecule, could have established and maintained these senescence messaging factors since the cells harbouring HPV episomes are known to regulate several DDR-activated proteins such as ATR (ataxia telangiectasia and Rad3-related protein), ATM (ataxia telangiectasia mutated), Chk1 (checkpoint kinase 1), Chk2, BRCA1 (breast cancer 1, early onset) and Nbs1 (nibrin) [48–50] for the viral existence and propagation. Another novel observation, in the present study, is the stabilization of senescent markers HMGA1 and HMGB1 in cells transfected with E2 and treated with TNF-α. HMGA1 and HMGB1, members of non-histone chromosomal proteins, have been recently demonstrated to play important roles in SASP [51,52]. HMGA1 cooperates with p16INK4a and p53 in a mutually reinforcing manner to induce and promote SA heterochromatic factors, accelerate senescence at physiologically relevant levels and repress cell-cycle genes [51] whereas HMGB1 is relocalized to the cytosol of cancer tissues [53]. Also, in breast cancer cells, HMGA1 binds Bcl2 promoter modulating p53-mediated transcriptional activity on Bcl2 promoter and repressing p53 apoptotic function [54]. Senescent cells secrete HMGB1 promoting secretion of inflammatory cytokines (TNFα, IL-1β and IL-6) by macrophages [55]. Altered HMGB1 expression induced senescence and secretion of IL-6, whereas its depletion, not overexpression, suppressed NF-κB activity and IL-6 secretion [56]. Most importantly, both HMGA1 and HMGB1 cooperate with NF-κB to bind DNA, form enhanceosome and regulate its downstream targets [57–60]. We conclude that the potentiation of NF-κB by E2 in the presence of TNF-α induction and the consequent increase in p53, Bcl2 and IL-6 is in line with the observed increase in expression and stabilization of two independent senescent markers, HMGA1 and HMGB1.

Though the present study did not assess the role of DDR proteins in E2-mediated senescence, it is assumed that the ability of HPV-E2 to induce DNA damage as previously known, together with TNF-α might play a role in imparting such SA secretory pattern and hence this would open new avenues for future studies. According to the present results, HPV16 E2 protein could contribute to the HPV oncogenic potential in presence of TNF-α by synergistically activating NF-κB and by enhancing the expression of cell survival factors. It is imperative to attribute an important role to E2 in cancerous lesions in sustaining tumorigenesis and to understand the role of chronic inflammation in sustaining and augmenting this damage is vital in future therapies for cervical cancer patients.

## Online data

Supplementary data

## References

[1] Thomison J., Thomas L. K., Shroyer K. R. (2008). Human papillomavirus: molecular and cytologic/histologic aspects related to cervical intraepithelial neoplasia and carcinoma. Hum. Pathol..

[2] Hanning J.E., Saini H.K., Murray M.J., Caffarel M.M., van Dongen S., Ward D., Barker E.M., Scarpini C.G., Groves I.J., Stanley M.A. (2013). Depletion of HPV16 early genes induces autophagy and senescence in a cervical carcinogenesis model, regardless of viral physical state. J. Pathol..

[3] Frattini M.G., Hurst S.D., Lim H.B., Swaminathan S., Laimins L.A. (1997). Abrogation of a mitotic checkpoint by E2 proteins from oncogenic human papillomaviruses correlates with increased turnover of the p53 tumor suppressor protein. EMBO J..

[4] Kalantari M., Karlsen F., Kristensen G., Holm R., Hagmar B., Johansson B. (1998). Disruption of the E1 and E2 reading frames of HPV 16 in cervical carcinoma is associated with poor prognosis. Int. J. Gynecol. Pathol..

[5] Goodwin E.C., Naeger L.K., Breiding D.E., Androphy E.J., DiMaio D. (1998). Transactivation-competent bovine papillomavirus E2 protein is specifically required for efficient repression of human papillomavirus oncogene expression and for acute growth inhibition of cervical carcinoma cell lines. J. Virol..

[6] Thierry F., Demeret C. (2008). Direct activation of caspase 8 by the proapoptotic E2 protein of HPV18 independent of adaptor proteins. Cell Death Differ..

[7] Harris S.L., Levine A.J. (2005). The p53 pathway: positive and negative feedback loops. Oncogene.

[8] Trottier H., Ferreira S., Thomann P., Costa M.C., Sobrinho J.S., Prado J.C., Rohan T.E., Villa L.L., Franco E.L. (2010). Human papillomavirus infection and reinfection in adult women: the role of sexual activity and natural immunity. Cancer Res..

[9] Wei L., Gravitt P.E., Song H., Maldonado A.M., Ozbun M.A. (2009). Nitric oxide induces early viral transcription coincident with increased DNA damage and mutation rates in human papillomavirus-infected cells. Cancer Res..

[10] Stanley M. (2006). Immune responses to human papillomavirus. Vaccine.

[11] Vieira K.B., Goldstein D.J., Villa L.L. (1996). Tumor necrosis factor alpha interferes with the cell cycle of normal and papillomavirus-immortalized human keratinocytes. Cancer Res..

[12] Filippova M., Song H., Connolly J.L., Dermody T.S., Duerksen-Hughes P.J. (2002). The human papillomavirus 16 E6 protein binds to tumor necrosis factor (TNF) R1 and protects cells from TNF-induced apoptosis. J. Biol. Chem..

[13] Villa L.L., Vieira K.B., Pei X.F., Schlegel R. (1992). Differential effect of tumor necrosis factor on proliferation of primary human keratinocytes and cell lines containing human papillomavirus types 16 and 18. Mol. Carcinog..

[14] Boulabiar M., Boubaker S., Favre M., Demeret C. (2011). Keratinocyte sensitization to TNF-induced NF-{kappa}B activation by the E2 regulatory protein of human papillomaviruses. J. Gen. Virol..

[15] Prabhavathy D., Prabhakar B.N., Karunagaran D. (2014). HPV16 E2-mediated potentiation of NF-kappaB activation induced by TNF-alpha involves parallel activation of STAT3 with a reduction in E2-induced apoptosis. Mol. Cell. Biochem..

[16] Janssens S., Tschopp J. (2006). Signals from within: the DNA-damage-induced NF-kappaB response. Cell Death Differ..

[17] Wells S.I., Francis D.A., Karpova A.Y., Dowhanick J.J., Benson J.D., Howley P.M. (2000). Papillomavirus E2 induces senescence in HPV-positive cells via pRB- and p21(CIP)-dependent pathways. EMBO J..

[18] Desaintes C., Demeret C., Goyat S., Yaniv M., Thierry F. (1997). Expression of the papillomavirus E2 protein in HeLa cells leads to apoptosis. EMBO J..

[19] Goodwin E.C., Yang E., Lee C.J., Lee H.W., DiMaio D., Hwang E.S. (2000). Rapid induction of senescence in human cervical carcinoma cells. Proc. Natl. Acad. Sci. U.S.A..

[20] Hwang E.S., Naeger L.K., DiMaio D. (1996). Activation of the endogenous p53 growth inhibitory pathway in HeLa cervical carcinoma cells by expression of the bovine papillomavirus E2 gene. Oncogene.

[21] Gary R.K., Kindell S.M. (2005). Quantitative assay of senescence-associated beta-galactosidase activity in mammalian cell extracts. Anal. Biochem..

[22] Gaiotti D., Chung J., Iglesias M., Nees M., Baker P.D., Evans C.H., Woodworth C.D. (2000). Tumor necrosis factor-alpha promotes human papillomavirus (HPV) E6/E7 RNA expression and cyclin-dependent kinase activity in HPV-immortalized keratinocytes by a ras-dependent pathway. Mol. Carcinog..

[23] Tommasino M., Accardi R., Caldeira S., Dong W., Malanchi I., Smet A., Zehbe I. (2003). The role of TP53 in Cervical carcinogenesis. Hum. Mutat..

[24] Gonzalez S.L., Stremlau M., He X., Basile J.R., Munger K. (2001). Degradation of the retinoblastoma tumor suppressor by the human papillomavirus type 16 E7 oncoprotein is important for functional inactivation and is separable from proteasomal degradation of E7. J. Virol..

[25] Bhattacharya S., Ray R.M., Johnson L.R. (2005). STAT3-mediated transcription of Bcl-2, Mcl-1 and c-IAP2 prevents apoptosis in polyamine-depleted cells. Biochem. J..

[26] Grivennikov S.I., Karin M. (2010). Dangerous liaisons: STAT3 and NF-kappaB collaboration and crosstalk in cancer. Cytokine Growth Factor Rev..

[27] Bellanger S., Blachon S., Mechali F., Bonne-Andrea C., Thierry F. (2005). High-risk but not low-risk HPV E2 proteins bind to the APC activators Cdh1 and Cdc20 and cause genomic instability. Cell Cycle.

[28] Scheffner M., Huibregtse J.M., Vierstra R.D., Howley P.M. (1993). The HPV-16 E6 and E6-AP complex functions as a ubiquitin-protein ligase in the ubiquitination of p53. Cell.

[29] Webster K., Parish J., Pandya M., Stern P.L., Clarke A.R., Gaston K. (2000). The human papillomavirus (HPV) 16 E2 protein induces apoptosis in the absence of other HPV proteins and via a p53-dependent pathway. J. Biol. Chem..

[30] Parish J.L., Kowalczyk A., Chen H.T., Roeder G.E., Sessions R., Buckle M., Gaston K. (2006). E2 proteins from high- and low-risk human papillomavirus types differ in their ability to bind p53 and induce apoptotic cell death. J. Virol..

[31] el-Deiry W.S., Tokino T., Velculescu V.E., Levy D.B., Parsons R., Trent J.M., Lin D., Mercer W.E., Kinzler K.W., Vogelstein B. (1993). WAF1, a potential mediator of p53 tumor suppression. Cell.

[32] Basile J.R., Eichten A., Zacny V., Munger K. (2003). NF-kappaB-mediated induction of p21(Cip1/Waf1) by tumor necrosis factor alpha induces growth arrest and cytoprotection in normal human keratinocytes. Mol. Cancer Res..

[33] Basile J.R., Zacny V., Munger K. (2001). The cytokines tumor necrosis factor-alpha (TNF-alpha) and TNF-related apoptosis-inducing ligand differentially modulate proliferation and apoptotic pathways in human keratinocytes expressing the human papillomavirus-16 E7 oncoprotein. J. Biol. Chem..

[34] DeFilippis R.A., Goodwin E.C., Wu L., DiMaio D. (2003). Endogenous human papillomavirus E6 and E7 proteins differentially regulate proliferation, senescence, and apoptosis in HeLa cervical carcinoma cells. J. Virol..

[35] Sima N., Wang S., Wang W., Kong D., Xu Q., Tian X., Luo A., Zhou J., Xu G., Meng L. (2007). Antisense targeting human papillomavirus type 16 E6 and E7 genes contributes to apoptosis and senescence in SiHa cervical carcinoma cells. Gynecol. Oncol..

[36] Klefstrom J., Arighi E., Littlewood T., Jaattela M., Saksela E., Evan G.I., Alitalo K. (1997). Induction of TNF-sensitive cellular phenotype by c-Myc involves p53 and impaired NF-kappaB activation. EMBO J..

[37] Malejczyk J., Malejczyk M., Majewski S., Breitburd F., Luger T.A., Jablonska S., Orth G. (1994). Increased tumorigenicity of human keratinocytes harboring human papillomavirus type 16 is associated with resistance to endogenous tumor necrosis factor-alpha-mediated growth limitation. Int. J. Cancer.

[38] Wu Z.H., Miyamoto S. (2008). Induction of a pro-apoptotic ATM-NF-kappaB pathway and its repression by ATR in response to replication stress. EMBO J..

[39] Ghosh A., Saginc G., Leow S.C., Khattar E., Shin E.M., Yan T.D., Wong M., Zhang Z., Li G., Sung W.K. (2012). Telomerase directly regulates NF-kappaB-dependent transcription. Nat. Cell Biol..

[40] Low K.C., Tergaonkar V. (2013). Telomerase: central regulator of all of the hallmarks of cancer. Trends Biochem. Sci..

[41] You Z., Madrid L.V., Saims D., Sedivy J., Wang C.Y. (2002). c-Myc sensitizes cells to tumor necrosis factor-mediated apoptosis by inhibiting nuclear factor kappa B transactivation. J. Biol. Chem..

[42] Duerksen-Hughes P.J., Yang J., Schwartz S.B. (1999). HPV 16 E6 blocks TNF-mediated apoptosis in mouse fibroblast LM cells. Virology.

[43] Tamatani M., Che Y.H., Matsuzaki H., Ogawa S., Okado H., Miyake S., Mizuno T., Tohyama M. (1999). Tumor necrosis factor induces Bcl-2 and Bcl-x expression through NFkappaB activation in primary hippocampal neurons. J. Biol. Chem..

[44] Salminen A., Kauppinen A., Kaarniranta K. (2012). Emerging role of NF-kappaB signaling in the induction of senescence-associated secretory phenotype (SASP). Cell. Signal..

[45] Coppe J.P., Desprez P.Y., Krtolica A., Campisi J. (2010). The senescence-associated secretory phenotype: the dark side of tumor suppression. Ann. Rev. Pathol..

[46] Duensing S., Munger K. (2004). Mechanisms of genomic instability in human cancer: insights from studies with human papillomavirus oncoproteins. Int. J. Cancer..

[47] Hopman A.H., Smedts F., Dignef W., Ummelen M., Sonke G., Mravunac M., Vooijs G.P., Speel E.J., Ramaekers F.C. (2004). Transition of high-grade cervical intraepithelial neoplasia to micro-invasive carcinoma is characterized by integration of HPV 16/18 and numerical chromosome abnormalities. J. Pathol..

[48] Edwards T.G., Vidmar T.J., Koeller K., Bashkin J.K., Fisher C. (2013). DNA damage repair genes controlling human papillomavirus (HPV) episome levels under conditions of stability and extreme instability. PLoS One.

[49] Moody C.A., Laimins L.A. (2009). Human papillomaviruses activate the ATM DNA damage pathway for viral genome amplification upon differentiation. PLoS Pathog.

[50] Edwards T.G., Helmus M.J., Koeller K., Bashkin J.K., Fisher C. (2013). Human papillomavirus episome stability is reduced by aphidicolin and controlled by DNA damage response pathways. J. Virol..

[51] Narita M., Narita M., Krizhanovsky V., Nunez S., Chicas A., Hearn S.A., Myers M.P., Lowe S.W. (2006). A novel role for high-mobility group a proteins in cellular senescence and heterochromatin formation. Cell.

[52] Zhang R., Chen W., Adams P.D. (2007). Molecular dissection of formation of senescence-associated heterochromatin foci. Mol. Cell Biol..

[53] Livesey K.M., Kang R., Vernon P., Buchser W., Loughran P., Watkins S.C., Zhang L., Manfredi J.J., Zeh H.J. (2012). p53/HMGB1 complexes regulate autophagy and apoptosis. Cancer Res..

[54] Esposito F., Tornincasa M., Chieffi P., De Martino I., Pierantoni G.M., Fusco A. (2010). High-mobility group A1 proteins regulate p53-mediated transcription of Bcl-2 gene. Cancer Res..

[55] Davalos A.R., Coppe J.P., Campisi J., Desprez P.Y. (2010). Senescent cells as a source of inflammatory factors for tumor progression. Cancer Metastasis Rev..

[56] Davalos A.R., Kawahara M., Malhotra G.K., Schaum N., Huang J., Ved U., Beausejour C.M., Coppe J.P., Rodier F., Campisi J. (2013). p53-dependent release of Alarmin HMGB1 is a central mediator of senescent phenotypes. J. Cell Biol..

[57] Thanos D., Maniatis T. (1992). The high mobility group protein HMG I(Y) is required for NF-kappa B-dependent virus induction of the human IFN-beta gene. Cell.

[58] Zhang X.M., Verdine G.L. (1999). A small region in HMG I(Y) is critical for cooperation with NF-kappaB on DNA. J. Biol. Chem..

[59] Resar L.M. (2010). The high mobility group A1 gene: transforming inflammatory signals into cancer?. Cancer Res..

[60] Agresti A., Lupo R., Bianchi M.E., Muller S. (2003). HMGB1 interacts differentially with members of the Rel family of transcription factors. Biochem. Biophys. Res. Commun..

