# Supplementary material for: Re-expression of HPV16 E2 in SiHa (human cervical cancer) cells potentiates NF-κB activation induced by TNF-α concurrently increasing senescence and survival
Source: Biosci Rep. 2015 Feb 25;35(1):e00175. doi: 10.1042/BSR20140160 (PMC4340273; doi:10.1042/BSR20140160)
Supplement: Supplementary data [file bsr035e175ntsadd.pdf]

Supplementary Table 1 List of primers used for qPCR and their corresponding annealing temperatures. The cycling conditions were: 95˚C for 10 min, followed by 40 cycles of 95˚C for 15 sec, Tm for 20 sec and 62˚C for 15 sec.

**GENE**		**SEQUENCE (5'-3')**	**Annealing temperature**
cyc D1	Forward	CCGTCCATGCGGAAGATC	62ºC
	Reverse	ATGGCCAGCGGGAAGAC	
c-Myc	Forward	TCAAGAGGTGCCACGTCTCC	62ºC
	Reverse	TCTTGGCAGCAGGATAGTCCTT	
survivin	Forward	AGAACTGGCCCTTCTTGGGGTC	60ºC
	Reverse	CTTTTTATGTTCCTCTATGGGGTC	
hTERT	Forward	AAGTTCCTGCACTGGCTGAT	60ºC
	Reverse	CACGACGTAGTCCATGTTC	
HPV16 E6	Forward	TGCAATGTTTCAGGACCCA	60ºC
	Reverse	CATGTATAGTTGTTTGCAGCTCTGT	
IL-6	Forward	TACCCCCAGGAGAAGATTCC	60ºC
	Reverse	GCCATCTTTGGAAGGTTCAG	
IL-8	Forward	AGGTGCAGTTTTGCCAAGGA	60ºC
	Reverse	TTTCTGTGTTGGCGCAGTGT	
β-actin	Forward	TCATGAAGATCCTCACCGAG	58ºC
	Reverse	TTGCCAATGGTGATGACCTG	
